# An open natural language processing (NLP) framework for EHR-based clinical research: a case demonstration using the National COVID Cohort Collaborative (N3C)

**DOI:** 10.1093/jamia/ocad134

**Published:** 2023-08-09

**Authors:** Sijia Liu, Andrew Wen, Liwei Wang, Huan He, Sunyang Fu, Robert Miller, Andrew Williams, Daniel Harris, Ramakanth Kavuluru, Mei Liu, Noor Abu-el-Rub, Dalton Schutte, Rui Zhang, Masoud Rouhizadeh, John D Osborne, Yongqun He, Umit Topaloglu, Stephanie S Hong, Joel H Saltz, Thomas Schaffter, Emily Pfaff, Christopher G Chute, Tim Duong, Melissa A Haendel, Rafael Fuentes, Peter Szolovits, Hua Xu, Hongfang Liu

**Affiliations:** Department of Artificial Intelligence and Informatics, Mayo Clinic, Rochester, Minnesota, USA; Department of Artificial Intelligence and Informatics, Mayo Clinic, Rochester, Minnesota, USA; Department of Artificial Intelligence and Informatics, Mayo Clinic, Rochester, Minnesota, USA; Department of Artificial Intelligence and Informatics, Mayo Clinic, Rochester, Minnesota, USA; Department of Artificial Intelligence and Informatics, Mayo Clinic, Rochester, Minnesota, USA; Tufts Clinical and Translational Science Institute, Tufts Medical Center, Boston, Massachusetts, USA; Tufts Clinical and Translational Science Institute, Tufts Medical Center, Boston, Massachusetts, USA; Department of Internal Medicine, University of Kentucky, Lexington, Kentucky, USA; Department of Internal Medicine, University of Kentucky, Lexington, Kentucky, USA; Department of Internal Medicine, University of Kansas Medical Center, Kansas City, Kansas, USA; Department of Internal Medicine, University of Kansas Medical Center, Kansas City, Kansas, USA; Department of Pharmaceutical Care & Health Systems, University of Minnesota at Twin Cities, Minneapolis, Minnesota, USA; Department of Pharmaceutical Care & Health Systems, University of Minnesota at Twin Cities, Minneapolis, Minnesota, USA; Department of Pharmaceutical Outcomes & Policy, University of Florida, Gainesville, Florida, USA; Department of Computer Science, University of Alabama at Birmingham, Birmingham, Alabama, USA; Department of Computational Medicine and Bioinformatics, University of Michigan Medical School, Ann Arbor, Michigan, USA; Department of Cancer Biology, Wake Forest School of Medicine, Winston-Salem, North Carolina, USA; Department of Medicine, Johns Hopkins University, Baltimore, Maryland, USA; Department of Biomedical Informatics, Stony Brook University, Stony Brook, New York, USA; Sage Bionetwork, Seattle, Washington, USA; Department of Medicine, University of North Carolina Chapel Hill, Chapel Hill, North Carolina, USA; Department of Medicine, Johns Hopkins University, Baltimore, Maryland, USA; Department of Radiology, Albert Einstein College of Medicine, Bronx, New York, USA; Center for Health AI, University of Colorado Anschutz Medical Campus, Denver, Colorado, USA; Alex Informatics, North Bethesda, Maryland, USA; Department of Electrical Engineering and Computer Science, Massachusetts Institute of Technology, Cambridge, Massachusetts, USA; School of Biomedical Informatics, University of Texas Health Science Center at Houston, Houston, Texas, USA; Department of Artificial Intelligence and Informatics, Mayo Clinic, Rochester, Minnesota, USA; School of Biomedical Informatics, University of Texas Health Science Center at Houston, Houston, Texas, USA

**Keywords:** electronic healthy records, natural language processing, federated learning, multi-institutional data annotation

## Abstract

Despite recent methodology advancements in clinical natural language processing (NLP), the adoption of clinical NLP models within the translational research community remains hindered by process heterogeneity and human factor variations. Concurrently, these factors also dramatically increase the difficulty in developing NLP models in multi-site settings, which is necessary for algorithm robustness and generalizability. Here, we reported on our experience developing an NLP solution for Coronavirus Disease 2019 (COVID-19) signs and symptom extraction in an open NLP framework from a subset of sites participating in the National COVID Cohort (N3C). We then empirically highlight the benefits of multi-site data for both symbolic and statistical methods, as well as highlight the need for federated annotation and evaluation to resolve several pitfalls encountered in the course of these efforts.

## BACKGROUND AND SIGNIFICANCE

Over the past decade, electronic health record (EHR) systems have been increasingly implemented across healthcare institutions. Large amounts of detailed longitudinal patient information have consequently been accumulated and made electronically available for analysis. One common challenge is the prevalence of clinical information embedded in clinical text,^1^ which commonly varies by institution in the practices of documenting impressions, findings, assessments, and care plans. Natural language processing (NLP) has demonstrated a great potential to extract information from text.^2^ However, NLP algorithms are mostly developed and applied in single-site settings.^3^ Therefore, many algorithms suffer from limited external validity and research inclusiveness. In comparison, multi-site studies typically generate more valid research findings due to larger sample sizes, greater representations of participant demographics (eg, age, gender, race, ethnicity, and social-economic status), and more diverse investigator expertise.^4–7^ Moreover, NLP algorithms developed at a single site tend to have poor generalizability when ported to other institutions.^8–11^ There is an urgent need to develop, evaluate, and deploy NLP solutions for multi-site studies.

Despite the need, widespread adoption of NLP solutions has faced several barriers, such as ETL (extract, transform, load) process heterogeneity between different sites and their EHR environments and human factor variations in gold standard corpus developments. Specifically, with respect to the ETL heterogeneity, variations in EHR system vendors, data infrastructure, and operations can lead to idiosyncratic approaches of clinical documentation, transformation, and representation.^12^^,^^13^ It typically requires some technical efforts to adapt external solutions locally.

For human factor variations, a key step before model development is corpus annotation. Annotation is the process of developing a gold standard by marking the occurrence of both task-defined sets of clinical information and their associated interpretative linguistic features (eg, subject, certainty, status). The creation of gold standard corpora require significant expenditure of domain expertise and time, as clinical experts regularly make decisions directly affecting the study cohort, annotation guideline, and task definitions.

To address these needs, we have previously developed the MedTagger NLP engine^14^ as part of the OHNLP Toolkit,^15^ a coordinated, transparent, and collaborative platform that promotes open team science collaboration in NLP algorithm development and evaluation through consensus building, process coordination, and best practice sharing.

## OBJECTIVE

In this communication, we aim to highlight the importance of multi-site data being used in algorithm development and identify the challenges of federated development and evaluation processes. We presented our experience developing an NLP solution for extracting Coronavirus Disease 2019 (COVID-19) signs and symptoms for the National COVID Cohort Consortium (N3C)^16–18^ via deployment of the OHNLP toolkit and examining the successes or failures of such.

## MATERIALS AND METHODS

Following the recommendation from the US Centers for Disease Control and Prevention (CDC) and Mayo Clinic, 20 signs and symptoms of COVID-19 were collected as a basic COVID-19 concept set. We then gathered additional lexical variants for each clinical concept from the CIDO^19^ and Human Phenotype Ontology (HPO).^20^

### NLP engines

Symbolic (rule-based, as opposed to statistical methods-based and/or deep learning-based) NLP processing is done through the MedTagger NLP engine^14^ deployed with SQL-based linkages to the OHDSI NOTE and NOTE_NLP tables for input and output, respectively. The reason we opt for a symbolic solution is due to its simplicity, transparency, and interpretability compared to many non-symbolic methods while maintaining fully deterministic outcomes based on the definition of the rules compared to other methods that rely on random initialization. For greater detail on the overall individual components of the framework supporting MedTagger’s deployment to facilitate multi-site adoption, please refer to the “Implementation Details” section of the Supplementary Appendix.

For comparative purposes, deep learning-based NLP processing was done via Bio+ClinicalBERT^21^ fine-tuned for the named entity recognition (NER) task with a sequence length of 512, batch size of 16, and epoch count of 100. Early stopping method was used to determine the optimal number of epoch and prevent over-fitting.

### Dataset and deidentification

Clinical notes from patients with positive COVID-19 test results were collected from 3 institutions: the Mayo Clinic, the University of Kentucky (UKen), and the University of Minnesota at Twin Cities (UMN). We filtered out notes that were not office visit notes (eg, nurse calls), had fewer than 1000 characters, and were authored more than 14 days prior to the date of the patient’s earliest positive COVID-19 test result.

As access to clinical narratives is limited due to privacy concerns stemming from the presence of protected health information within the text, dataset deidentification was a necessity. Consequently, a total of 369 clinical notes from these sites that met these criteria were randomly selected to be deidentified using the Notes Deidentification program developed by the Medical College of Wisconsin^22^ followed by manual review. The removed identifiers were replaced with programmatically added synthetic text (ie, resynthesis). The multi-site train/split details can be found in the Supplementary Appendix.

### Training of NLP models

Single-site symbolic model development was conducted by running the initial symbolic ruleset derived from public ontologies on the Mayo train dataset, identifying errors, and subsequently adding additional symbolic rulesets to address these errors.

Single-site deep learning model development consisted of fine-tuning the Bio+ClinicalBERT model on the Mayo train dataset, where the sample size for fine-tuning may not need to be too large.^23^^,^^24^

The symbolic model was refined with multi-site data by first running the single-site Mayo model on the additional training data sourced from non-Mayo sites. Errors were identified as corresponding to 8 distinct concepts, and appropriate symbolic rules were added to address these errors. (For a detailed tabular view of changes to the symbolic ruleset resulting from this multi-site fine-tuning, please refer to the “Ruleset Statistics” section of the Supplementary Appendix.)

Multi-site conversion of the deep learning-based model consisted of retraining the NER task fine-tuning on the combined multi-site train set.

### Train and test set gold standard annotation

Given the lack of resources at individual sites for creating benchmark annotations, the dataset was centrally gathered from the various participating sites at the Mayo Clinic after de-identification through the previously outlined framework.

The annotation process was completed by an annotation team from Mayo Clinic to generate the gold standard annotations on COVID-19 signs and symptoms. The annotation guideline was created and refined using the Mayo notes. The annotation team, consisting of a senior and a junior annotator, carried out the annotation effort. (The senior annotator is a registered nurse with more than 10-year experiences in text data annotation with clinical background. The junior annotator is a graduate student in health data analytics without clinical credential.) While waiting for de-identified notes from other sites, the training of the annotation team was primarily using notes from Mayo Clinic. The inter annotator agreement (IAA) was calculated after annotation and corresponding discrepancies were resolved by discussions between the 2 annotators led by the lead to generate a final gold standard dataset. This final adjudicated corpus was used for our experiment on multi-site NLP algorithm development and evaluation.

### Evaluation

Evaluation was done using the precision, recall, and F1-score metrics. These metrics were separately calculated for the symbolic and deep learning-based models in both single- and multi-site settings. For more details on the specific definitions involved in evaluation, please refer to the “Evaluation Method” section in the Supplementary Appendix.

## RESULTS

Corpus statistics, including the number of annotations, site-specific, and overall micro-average F1-score IAA for each of the 20 concepts of interest can be found in Table 1. The F1-score IAA of the annotated corpus was 0.686 for Mayo, 0.373 for UKen, and 0.521 for UMN.

**Table 1.** Annotation corpora statistics

Concepts	Mayo (313 notes)		UKen (20 notes)		UMN (36 notes)		Overall IAA
	Instance of concepts	IAA	Instance of concepts	IAA	Instance of concepts	IAA	
Abdominal_pain	59	0.768	2	0.667	3	0.667	0.762
Chest_pain	62	0.718	2	0.667	11	0.200	0.672
Chill	51	0.719	6	0	6	0.727	0.679
Cough	104	0.791	14	0.522	43	0.691	0.746
Cyanosis	9	0.143	4	0	17	0	0.065
Delirium	38	0.280	2	0	10	0	0.269
Diarrhea	92	0.731	5	0.667	11	0.824	0.736
Dyspnea	199	0.700	19	0.242	46	0.3	0.610
Fatigue	61	0.644	15	0.182	13	0.706	0.610
Fever	148	0.590	25	0.308	53	0.406	0.522
Headache	43	0.811	6	0.286	15	0.609	0.731
Hypersomnia	6	0.182	0	0	14	0	0.087
Loss_of_appetite	41	0.406	2	0	4	0.250	0.378
Loss_of_smell	23	0.683	4	0.333	6	0.909	0.690
Loss_of_taste	19	0.686	2	0	5	0.889	0.681
Myalgia	21	0.647	6	0.286	8	0.615	0.593
Nasal_obstruction	16	0.235	6	0	14	0.556	0.359
Nausea	87	0.681	7	0.833	10	0.714	0.695
Sore_throat	16	0.800	4	0.75	17	0.692	0.750
Vomiting	86	0.700	6	0.75	14	0.667	0.698

*Note*: The instance count of each concept in each site.

Mayo: Mayo Clinic; UKen: University of Kentucky; UMN: University at Minnesota.

A comparison of the rule-based symbolic and deep learning-based NLP in single-site versus multi-site settings on the named entity recognition task (we considered partial matches as correct predictions in the experiments) with their Bootstrap Confidence Intervals^25^ can be found in Table 2. The performance of the multi-site NLP algorithm was generally superior to that of the single-site NLP algorithm with a degrading trend when porting the algorithm from Mayo to other sites. (For a detailed error analysis of NLP vs gold standard mismatches, please refer to the “Error Analysis” section of the Supplementary Appendix.)

**Table 2.** A comparison of a symbolic NLP model with a deep learning model based on the span level performance.

Algorithm type	Training data type	Dataset	Macro precision	Macro recall	Macro F1	Micro precision	Micro recall	Micro-F1 (95% CI^a^)
Rule-based	Single-site	Mayo	0.869	0.842	0.844	0.855	0.899	0.876 (0.852, 0.897)
		UKen	0.698	0.705	0.678	0.647	0.755	0.697 (0.631, 0.751)
		UMN	0.718	0.778	0.724	0.688	0.833	0.754 (0.717, 0.795)
	Multi-site	Mayo	0.87	0.863	0.853	0.863	0.908	0.884 (0.859, 0.905)
		UKen^b^	0.805	0.893	0.788	0.696	0.859	0.769 (0.687, 0.834)
		UMN^b^	0.828	0.882	0.829	0.718	0.918	0.806 (0.761, 0.849)
Deep learning-based	Single-site	Mayo	0.718	0.726	0.688	0.563	0.783	0.655 (0.578, 0.695)
		UKen	0.418	0.478	0.414	0.381	0.528	0.442 (0.367, 0.506)
		UMN	0.534	0.598	0.52	0.496	0.662	0.567 (0.519, 0.614)
	Multi-site	Mayo	0.718	0.726	0.688	0.563	0.783	0.655 (0.574, 0.695)
		UKen	0.43	0.467	0.412	0.386	0.677	0.492 (0.402, 0.579)
		UMN	0.703	0.718	0.671	0.578	0.711	0.637 (0.578, 0.695)

Mayo: Mayo Clinic; UKen: University of Kentucky; UMN: University at Minnesota.

a Bootstrap confidence interval.

b The combination of train and test sets were used for testing.

## DISCUSSION

In this study, we highlight the importance of using multi-site data for algorithm development and explore the challenges of the development and evaluation of NLP algorithms in single- and multi-site settings. Our experimental results support the need for multi-site development of NLP algorithms: regardless of the approach used, models refined using data from multiple sites showed a moderate-high improvement in generalizability in multi-site settings. Due to the limitation of data acquisition from outside sites, sample sizes for fine-tuning deep learning model and corresponding performance were low, which highlights the advantage of symbolic systems. However, our experience here also clearly highlights 2 additional bottlenecks in the overall workflow that would also be better served being done in a federated manner.

Firstly, due to resource constraints, the annotation process was centralized at Mayo as opposed to in a federated manner. Several pragmatic implementation challenges were discovered and may impact the intermediate and final NLP results. We observed that IAA varies between the 3 sites even though annotators had been trained using de-identified Mayo notes (0.686 F1-score for Mayo, 0.521 for UMN, and 0.373 for UKen). This was caused by several factors: (1) central collection of the text data itself took 4 months as each site needs to complete de-identification before sharing data; (2) as the annotation training began when Mayo data was first collected, it was difficult for annotators to remain consistent throughout the 4-month text collection process; and (3) the shared deidentified datasets were usually small. As a result, external notes were not used in the annotator training. The high variation in IAA illustrates that multi-site participation in NLP development was insufficient.

Secondly, our experiment shows the need for document annotation and evaluation itself to be a federated, site-local process given the lengthy process involved in procuring the limited multi-site dataset centrally. Site-specific factors such as documentation intention, data capture method, and document structure also impacted the annotation accuracy in this case study. These site-specific factors posed a challenge not only for annotation but also for the NLP algorithm development. In some cases, it was also ambiguous to the annotators that if a certain condition is related to COVID when adverse events and indication of treatment appeared. Federated annotation and evaluation would allow for these factors to be systematically incorporated by local expertise during the annotation process.

Beyond the question of centralized versus federated annotation and evaluation, the inconsistencies in definitions during error analysis highlights the need for a systematic framework for NLP error analysis as well as common definitions for the various heterogenous error types.

Our experimental findings also have several implications with respect to symbolic versus statistical models and ease of federated execution. Since symbolic NLP models are transparent to humans, the models can be shared without the risk of embedding personally identifiable information (PII) into models.

Conversely, the deidentification and data centralization process that our experimentation identifies as an undesirable bottleneck is currently difficult to avoid for statistical methods. Statistical model weights are considered to be possible PII leakage points for several participating institutions. The difficulty in centrally procuring deidentified data inherently conflicts with the need for a reasonably sized labeled dataset from multiple sites to build statistical NLP models. In our study, when comparing our symbolic NLP models with a fine-tuned Bio+ClinicalBERT model for the same task, we observed that the symbolic NLP models significantly outperform the deep learning models for concept extraction tasks. This is mainly due to small labeled dataset size. Similarly, the performance gain from multi-site fine-tuning is not as large as that with symbolic methods due to similar reasons. These results are consistent with many other application-specific information extraction tasks.^3^^,^^26^ While there have been promising advances in the federated statistical training space, such methods would still incur substantial overhead in the need for institutional review/approval relative to symbolic methods. Addressing these identified issues (ie, the need for federated annotation and evaluation, inconsistent error analysis) has been prioritized for further development of the OHNLP toolkit. We will report on our developed solutions to these issues as part of future work.

## Supplementary Material

ocad134_Supplementary_Data

## Data Availability

A detailed annotation guideline outlining the goals of the NLP task and how the corpora were annotated can be found at https://github.com/OHNLP/N3C-NLP-Documentation/wiki/Annotation-guideline-for-COVID-19-concepts. The sample deidentified synthetic corpus used as part of this study can be found at https://github.com/OHNLP/N3C-NLP-Documentation/blob/master/n3c_omop_sample.csv.

## CODE AVAILABILITY STATEMENT

Framework components can be found on GitHub:

- ETL Pipeline: https://github.com/OHNLP/Backbone
- NLP Implementation: https://github.com/OHNLP/MedTagger
- Web Rule Editor Front-end: https://github.com/OHNLP/OHNLPTK
- MedTator annotation tool: https://github.com/OHNLP/MedTator

The developed NLP ruleset can be found at https://github.com/OHNLP/covid19ruleset/tree/main/covid19.
