# Supplementary material for: An open natural language processing (NLP) framework for EHR-based clinical research: a case demonstration using the National COVID Cohort Collaborative (N3C)
Source: J Am Med Inform Assoc. 2023 Aug 9;30(12):2036–40. doi: 10.1093/jamia/ocad134 (PMC10654844; doi:10.1093/jamia/ocad134)
Supplement: ocad134_Supplementary_Data [file ocad134_supplementary_data.docx]

# Implementation Details

The framework is distributed as open-source software under the Apache 2.0 license via Github in three parts:

1. ETL Backbone with an example NLP engine at <https://github.com/OHNLP/Backbone>.
2. Process documentation at <https://github.com/OHNLP/N3C-NLP-Documentation>.
3. Open-source collaborative platform for developing NLP rulesets. We provide a demo homepage at <https://ohnlp4covid-dev.n3c.ncats.io/>

The demo homepage (Figure A.1 - <https://ohnlp4covid-dev.n3c.ncats.io/>) demonstrates the N3C NLP engine outputs on annotating clinical text using the baseline rulesets and dictionary. The annotations are from components of Sign/Symptom extractor, temporal information extractor and dictionary lookup extractor.

To further customize each model, the users can visit “Rule Editor” and the “Dictionary Builder” page (Figure A.). Figure A.3 provides an example of the rules editing interface with the baseline COVID-19 ruleset. The rulesets can be tested in real time by clicking the “Upload and test” button, where the rulesets will be uploaded, and the NLP engine will be generated for testing and debugging purposes. As a use case study, we also provide an example NLP project for extracting signs/symptoms related to COVID-19 that was developed as an example use case for this framework.

**
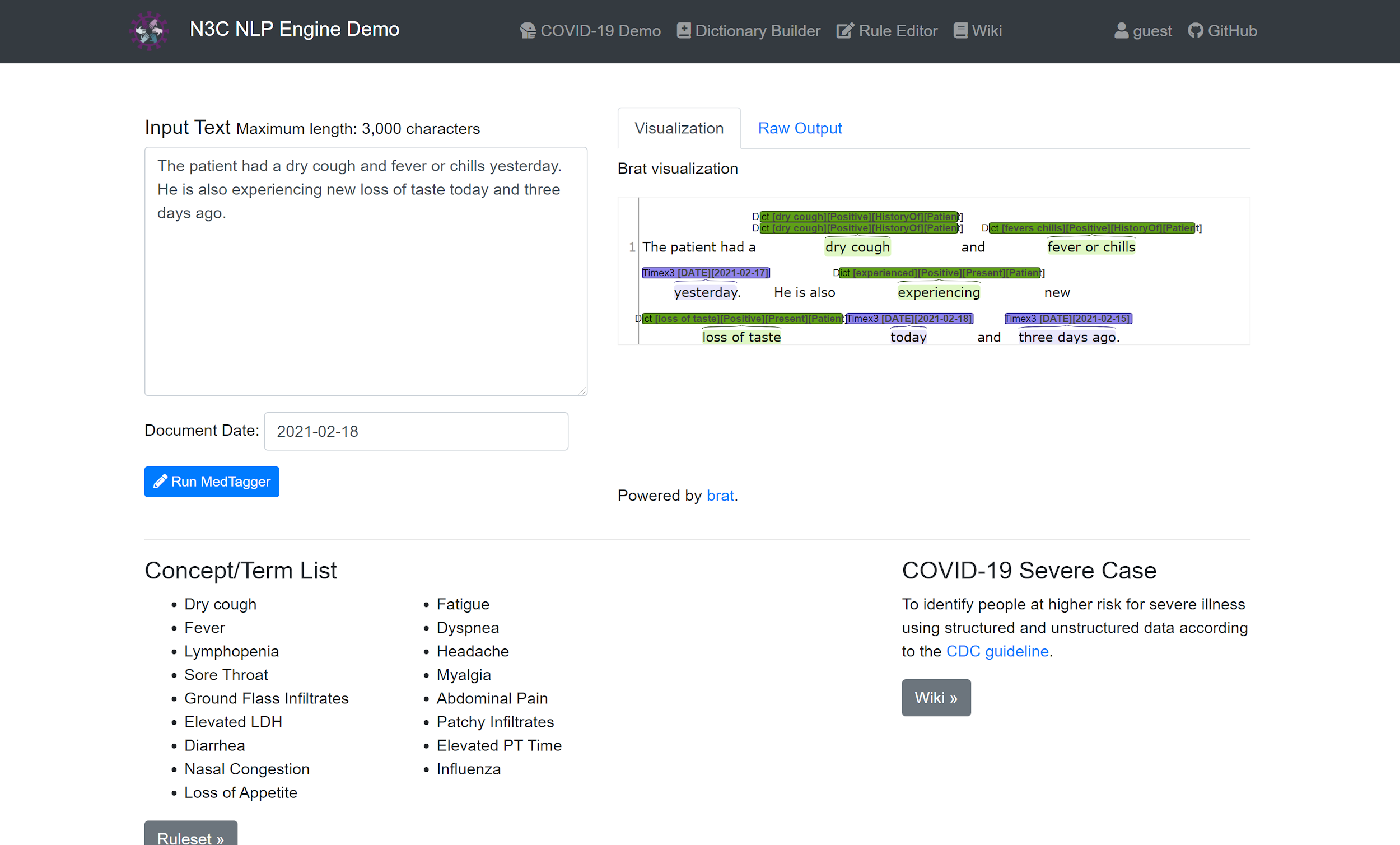
**

Figure A.1. Screenshot of the Web GUI homepage (<https://ohnlp4covid-dev.n3c.ncats.io/>)

The web GUI of the homepage consists of two components:

1. An input box for users to input text for evaluating the N3C NLP engine.
2. A visualized output by the N3C NLP engine with the baseline rulesets and dictionary. The output, including the recognized concepts, is highlighted with detailed attributes (e.g., certainty, normalized concept). In addition, users can also check the raw output in JSON (JavaScript Object Notation) format.
3. A list of concepts supported by the N3C NLP engine with the baseline rulesets and dictionary. Users can customize the concepts and definitions of this list by using our rule editor, which is shown in Figure A.2.


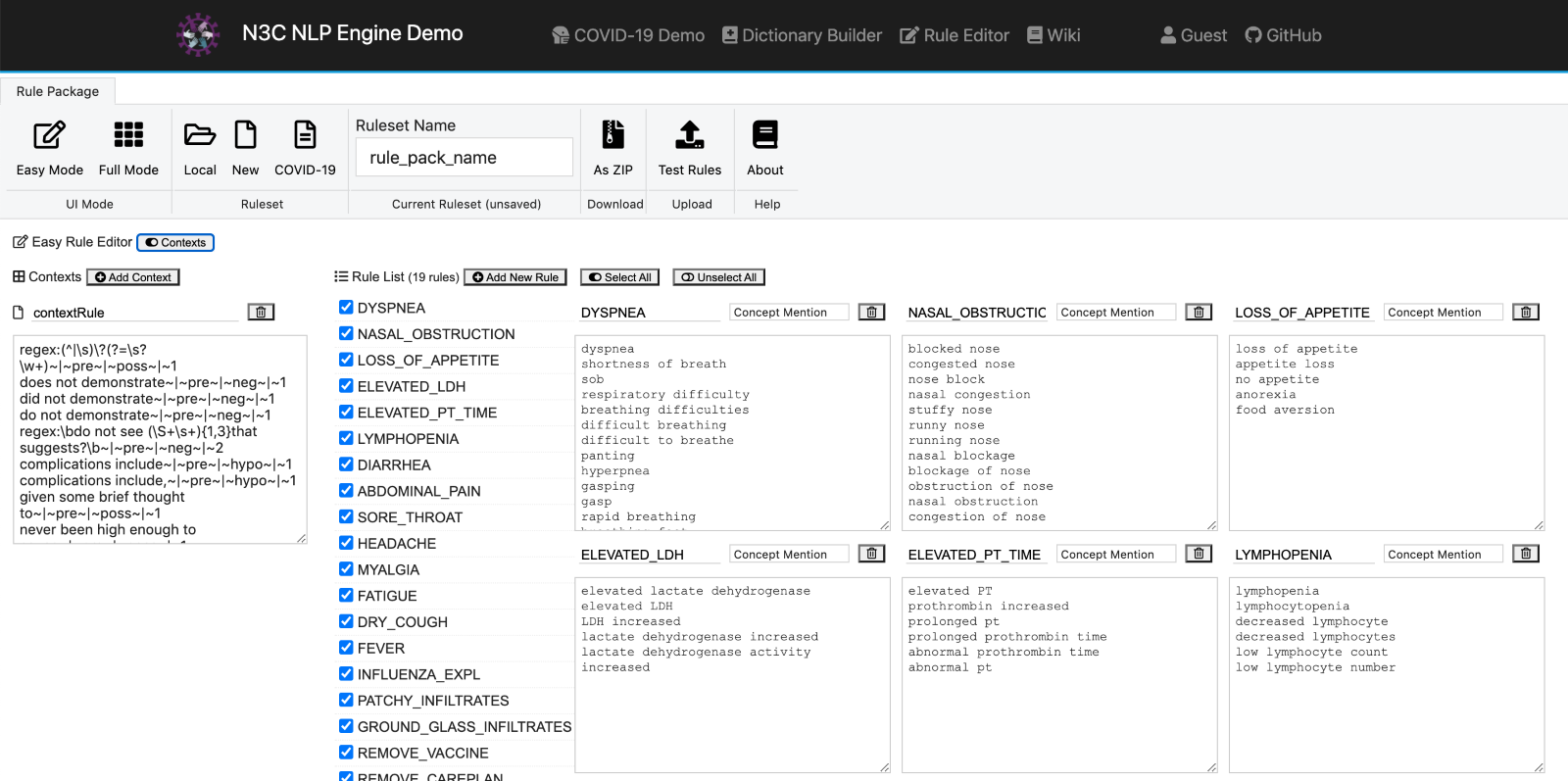


Figure A.2. Screenshot of the GUI of the rule editor.

The rule editor is designed to help users to create and modify rulesets that can be loaded by the N3C NLP engine. The GUI consists of three components:

1. Ribbon menu. This component provides several buttons to help users to load and save rulesets from the user's local disk or repository. Users can change the display mode to show/hide technical details of the user interface.
2. Context rule editor. This editor shows the contextual rules in a ruleset and allow users to modify the rules according to their own needs.
3. Concept list. Users can add/remove concepts to be identified in the N3C NLP engine.
4. Concept rule editor. The detailed rules of each concept are listed in an input box for users to review and revise. Each row in the input box represents a keyword or a regular expression to capture the concept mentioned in the text.

**
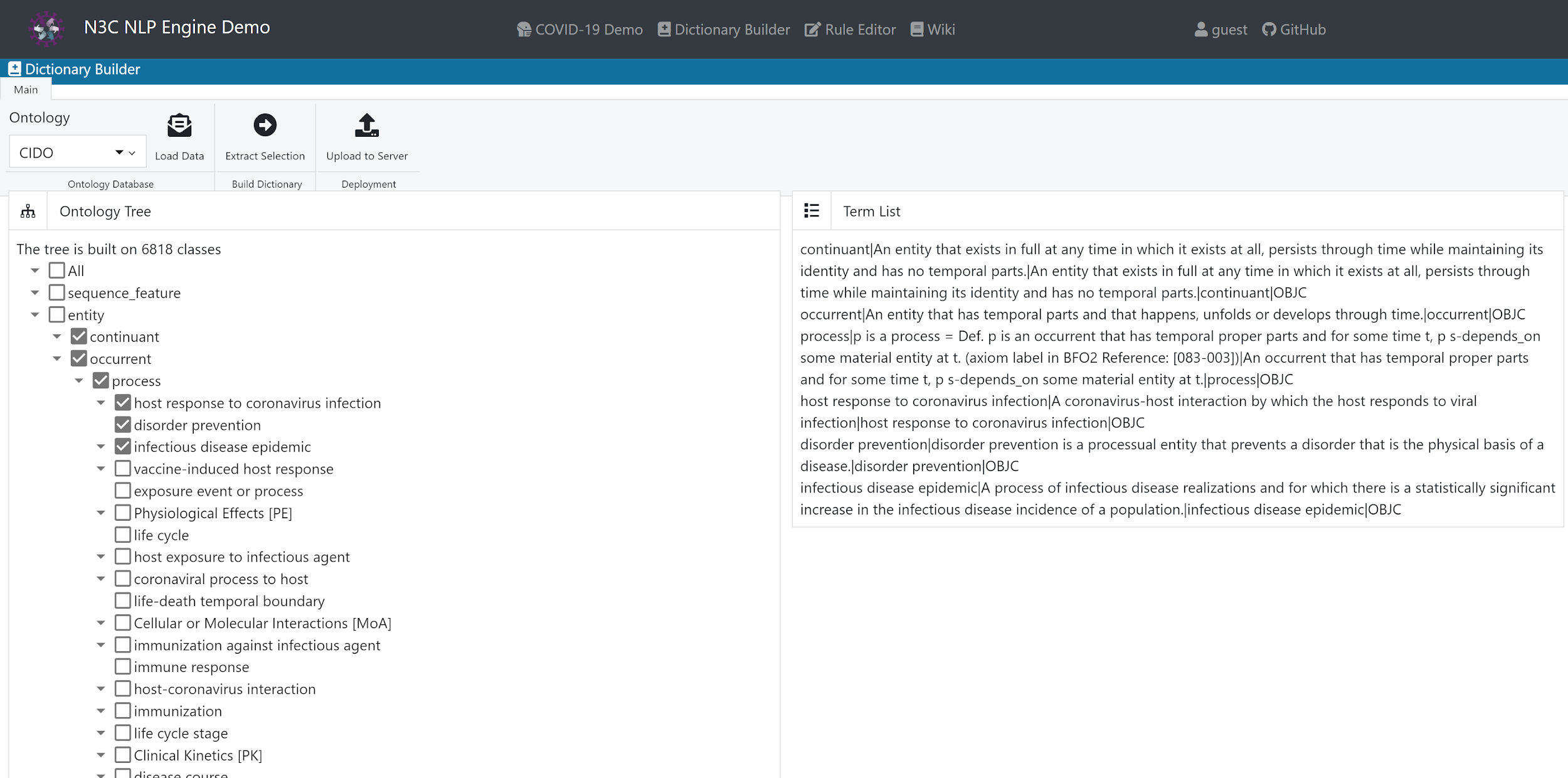
**

Figure A.3. Screenshot of the GUI of the dictionary editor.

The dictionary editor is designed to help users to browse pre-defined clinical concepts and create concept dictionaries that can be loaded by the N3C NLP engine. The GUI consists of three components:

1. Ribbon menu. This component provides several buttons to help users to load different clinical ontology datasets, such as CIDO (Coronavirus Infectious Disease) from BioPortal (<https://bioportal.bioontology.org/ontologies>).
2. Ontology tree viewer This viewer shows the selected ontology database in a tree-like table. Users can select the categories and terms in the tree to customize a dictionary.
3. Dictionary preview. The selected categories and terms are converted to our predefined dictionary format, which can be loaded into the N3C NLP engine.

# Evaluation Method

We evaluated the performance of single-site and multi-site algorithms using precision, recall, and F1-score for the annotated concept mentions, without and with certainty. Certainty is a categorical attribute of the concept mention for which possible values are [positive, negated, hypothetical, possible].

- **True positive (TP)**: For the mention-level evaluation without certainty, when there are overlaps between the gold standard mention span (start and end) and the NLP detected mention span while the concept type is the same, it is considered a true positive.
- **False negative (FN)**: If a concept mention exists in the gold standard annotation but not detected by the NLP algorithm, or spans overlap but the concept type is not matched, it is considered as a false negative.
- **False positive (FP)**: If a concept mention is detected by the algorithm but does not exist in the gold standard annotation, or spans overlap but the concept type is not matched, the concept is considered as a false positive.

For the mention-level span and certainty evaluation, certainty match needs to be considered when calculating TP, FN, and FP. Certainty match is determined as follows: if certainty matches, then it is considered a TP, if the certainty does not match, then it is considered a FP. For all system outputs without a matching type in the reference set, we count it as a FP (without a matched FN).

Micro- and macro- averages for each of the metrics of interest were calculated, where micro- refers to summing all the individual TP/FP/FN/TN counts across all classes (i.e., concept of interest to extract) before calculating the final metric, while macro- takes the average of the metric across all concepts.

The precision, recall, and F1-score are then calculated as follows. We further manually analyzed errors from multi-site algorithm mention-level evaluation without certainty.

$$Precision=\frac{TP}{TP+FP}$$

$$Recall=\frac{TP}{TP+FN}$$

$$F1=\frac{2*Precision*Recall}{Precision+Recall}$$

# Inter-annotator Agreement

The concept-level IAAs of each concept are summarized in Table A.1.

Table A.2. Concept-level IAA for three sites.

| **Concepts** | **IAA - Mayo** | **IAA - Uken** | **IAA - UMN** |
| --- | --- | --- | --- |
| Abdominal_pain | 0.767 | 0.667 | 0.667 |
| Chest_pain | 0.718 | 0.666 | 0.200 |
| Chill | 0.719 | 0 | 0.727 |
| Cough | 0.791 | 0.521 | 0.691 |
| Cyanosis | 0.142 | 0 | 0 |
| Delirium | 0.280 | 0 | 0 |
| Diarrhea | 0.730 | 0.666 | 0.823 |
| Dyspnea | 0.700 | 0.242 | 0.300 |
| Fatigue | 0.644 | 0.181 | 0.705 |
| Fever | 0.589 | 0.307 | 0.406 |
| Headache | 0.810 | 0.285 | 0.608 |
| Hypersomnia | 0.181 | 0 | 0 |
| Loss_of_appetite | 0.406 | 0 | 0.250 |
| Loss_of_smell | 0.682 | 0.333 | 0.910 |
| Loss_of_taste | 0.685 | 0 | 0.889 |
| Myalgia | 0.647 | 0.285 | 0.615 |
| Nasal_obstruction | 0.235 | 0 | 0.556 |
| Nausea | 0.680 | 0.833 | 0.714 |
| Sore_throat | 0.800 | 0.750 | 0.692 |
| Vomiting | 0.700 | 0.750 | 0.667 |

# Training/Testing split details

These 369 notes were then further divided into train/test sets for both single-site and multi-site settings. Specifically, the train/test split was 2:1 for notes originating from Mayo, and 1:1 for notes originating from other sites (stratified by originating site) due to low document set size. For single-site models, only the portion of the train set originating from the Mayo Clinic was used (n=206), while testing was separately conducted for each site. For multi-site models, the combined train set from all three sites is used (n=234) for training, and testing is separately conducted for each of the three sites using their respective test sets (Mayo n=107, UKen n=10, UMN n=18).

Specifically, in the single-site setting:

- Training: 206 (206 from Mayo)
- Testing: 163 (Mayo 107 + UKen 20 + UMN 36)

In the multi-site setting:

- Training: 234 (Mayo 206 + UKen 10 + UMN 18)
- Testing: 135 (Mayo 107 + UKen 10 + UMN 18)

# Error Analysis

Table A.3 shows the results of the error analysis for the three sites. For FP, major discrepancies between the NLP algorithm and the gold standard were due to the NLP algorithm extracting mentions that are not COVID-19 signs/symptoms of patients but for instruction/patient education, adverse events/indication of treatment, clinical goal/precaution, template, etc. It should be noted that gold standards were not always correct, and in some notes, it was hard to judge if the mentions were COVID signs/symptoms when symptoms are not appearing with COVID-19 or de-identified dates were inconsistent. For FN, reasons include NLP algorithm not complete, tokenization error due to de-identification process, template, and annotation errors.

Table A.3. Error analysis of the multi-site algorithm mention-level evaluation without certainty for three sites.

| **FP  Error Types** | **No. FP (%)** | | | **FN  Error Types** | **No. FN (%)** | | |
| --- | --- | --- | --- | --- | --- | --- | --- |
|  | **Mayo** | **UMN** | **UKen** |  | **Mayo** | **UMN** | **UKen** |
| Annotation error: missing annotation | 17 (26%) | 6 (12%) | 5 (21%) | NLP algorithm not complete | 21 (66%) | 11 (85%) | 7 (78%) |
| Hard to judge if are COVID signs/symptoms | 15 (23%) | 2 (4%) | 3 (13%) | Annotation error | 8 (25%) | 2 (15%) | 2 (22%) |
| Hypothetical: signs/symptoms from instruction/patient education, adverse events/indication of treatment, clinical goal/precaution, anesthesia plan, etc. | 26 (44%) | 37 (76%) | 13 (54%) | Tokenization error due to input format/de-identification software | 2 (6%) |  |  |
| Not COVID signs/symptoms - template | 5 (7%) | 4 (8%) | 2 (8%) | Template*/Form Text* | 1 (3%) |  |  |
| NLP algorithm not precise | 2 (3%) |  | 1 (4%) |  |  |  |  |

# Ruleset Statistics

Table A.4. shows a summary of rule development using single-site and multi-site data.

Table A.4. A summary for the single-site and multi-site rule development.

| **Concepts** | **No. Variants in original ruleset** | **Rule increase in multi-site version** |
| --- | --- | --- |
| Abdominal_pain | 16 | none |
| Chest_pain | 7 | none |
| Chill | 1 | none |
| Cough | 9 | none |
| Cyanosis | 5 | #UMN, add words between  bluish(\s+\S+){0,5}\s*lips  bluish(\s+\S+){0,5}\s*face |
| Delirium | 4 | //UK  deilirium |
| Diarrhea | 8 | none |
| Dyspnea | 25 | //UMN  WOB  breathing issue\w{0,3}  //UK  shortness of air |
| Fatigue | 14 | //UK  weak\w{0,5} |
| Fever | 15 | //UMN  (temp\|temperature)(\s+\S+){0,2}\s*(100\|101\|102\|103\|104\|105)(\.)?(0\|1\|2\|3\|4\|5\|6\|7\|8\|9)?  //UK  (100\|101\|102\|103\|104\|105\|106\|107\|108)(\.)?(  0\|1\|2\|3\|4\|5\|6\|7\|8\|9)?\s*(F\|fever)  (fever\|Tmax)(\W)?(\s+\S+){0,5}\s*(100\|101\|1  02\|103\|104\|105\|106\|107\|108)(\.)?(0\|1\|2\|3\|4\|  5\|6\|7\|8\|9)? |
| Headache | 9 | none |
| Hypersomnia | 5 | #UMN; UK  not(\s+\S+){0,5}\s*wak\w{0,5} up |
| Loss_of_appetite | 42 | //UMN  less hungry  //UK  decreased PO intake\w{0,2}  SOA |
| Loss_of_smell | 5 | none |
| Loss_of_taste | 6 | none |
| Myalgia | 12 | none |
| Nasal_obstruction | 23 | none |
| Nausea | 4 | none |
| Sore_throat | 10 | none |
| Vomiting | 4 | //UMN  not(\s+\S+){0,5}\s*keep fluids down |

# MINIMAR Statement

Table A.5 MINIMAR Statement of the study

| **Features** | **Description** |
| --- | --- |
| 1. Study population and setting |  |
| Population | All patients |
| Study setting | One hospital and two research medical centers |
| Data source | EHRs |
| Cohort selection | - Patients with research authorizations - Has positive COVID-19 results - Has clinical notes 2 weeks before AND 4 weeks after the lab order date of the first positive COVID-19 results |
| 1. Patient demographic characteristics | The patients are randomly selected and deidentified without statistics in demographics. Only the clinical texts are included in this study. We did not include patient demographics in our original data request and so were unable to perform a disaggregated analysis of performance by any demographic categories. |
| 1. Model architecture |  |
| Model output | The location of COVID-related concept mentions in the clinical text |
| Target user | Clinical analysts and researchers |
| Data splitting | The Training/Testing ratio is 2:1 at Mayo Clinic, 1:1 for the two other participating sites |
| Gold standard | Manually curated in the study |
| Model task | Named Entity Recognition (NER) |
| Model architecture | Symbolic (rule-based) method;  BERT/Transformer |
| Features | Raw text from clinical notes |
| Missingness | Not appliable |
| 4. Model evaluation |  |
| Optimization | Symbolic method: manual updates on the patterns  Deep Learning method: fine-tuning based on different experiment settings |
| Internal model validation | Not performed |
| External model validation | Cross-evaluated on the testing sets from other institutions |
| Transparency | Code and sample data available via Github |

# Acronyms

Table A.5. A summary of acronyms.

| API | Application Programming Interface |
| --- | --- |
| CDC | Centers for Disease Control and Prevention |
| CDM | Common Data Model |
| CIDO | Coronavirus Infectious Disease |
| CUI | Concept Unique Identifier |
| COVID-19 | Coronavirus disease 2019 |
| ETL | Extract, transform and load |
| HPO | Human Phenotype Ontology |
| IAA | Inter annotator agreement |
| MONDO | Mondo Disease Ontology |
| N3C | National COVID Cohort Consortium |
| NLP | Natural Language Processing |
| OMOP | Observational Medical Outcomes Partnership |
| UKen | University of Kentucky |
| UMLS | Unified Medical Language System |
| UMN | University of Minnesota at Twin Cities |
